# Editorial: Antiparasitic Activity and the Modes of Action of Natural Products and Traditional Medicines

**DOI:** 10.3389/fvets.2022.928643

**Published:** 2022-07-06

**Authors:** Xiaofei Shang, Annamaria Passantino, Gulnaz Ilgekbayeva, Jiyu Zhang

**Affiliations:** ^1^Key Laboratory of New Animal Drug Project, Key Laboratory of Veterinary Pharmaceutical Development of Ministry of Agriculture, Gansu Herbage and Livestock Environment Observation and Research Station, Lanzhou Institute of Husbandry and Pharmaceutical Sciences, Chinese Academy of Agricultural Sciences, Lanzhou, China; ^2^Department of Veterinary Sciences, University of Messina, Messina, Italy; ^3^Department of Biological Safety, Kazakh National Agrarian Research University, Almaty, Kazakhstan

**Keywords:** antiparasitic activity, natural product, traditional medicines, modes of action (MOA), lead compounds

Worldwide parasites pose a serious threat to the health of both humans and animals, leading to large economic losses. Currently, the treatment of parasitic diseases mainly relies on chemotherapy, and antiparasitic drugs represent the second largest segment of the global animal health market with 23% of the market share (1). During the past half century, significant treatments have been advancing, however, they remain a major threat to livestock farming and cause large deficits for the agricultural economy (1). In addition, the long-term use of chemical drugs has resulted in more serious problems, such as pesticide resistance, environmental contamination, environmental persistence, resurgence, and other side effects. These problems are not only destroying the healthy community structure of husbandry but also bring risks for humans (1, 2). Hence, more efforts are now being directed toward the global control of parasites in intensive livestock production.

In the past 50 years, traditional medicines and natural products have been considered an important alternative strategy for the sustainable management of parasitic diseases, and great progress has been achieved. Between January 1981 and September 2019, 20 antiparasitic agents were approved by the FDA, and nine agents (45%) were derived from natural products (3). Notably, the Nobel Prize in Physiology or Medicine 2015 was awarded jointly to William Campbell, Satoshi Omura, and Tu Youyou for their discoveries concerning a novel therapy against infections caused by roundworm parasites and Malaria, respectively (4). Considerable effort has been made to exploit the active compounds that occur naturally as secondary metabolites of plants, animals, and microorganisms, and some traditional medicines and natural products have been proven to have strong antiparasitic activity in both animals and humans *in vivo* and *in vitro*, especially for ectoparasites. After searching the web of science database, 284 papers related to anti-ectoparasite agents were published by *Veterinary Parasitology, Frontiers in Veterinary Sciences*, and other international journals worldwide from January 2015 to June 2020, and 204 papers (71.83%) aimed to find the active natural products or extracts from plants, 74 papers (26.06%) focused on essential oils (5).

Recently, along with the development of tremendous technologies in structural biology, computational chemistry, structure-based drug design, and multi-omics, coupled with enhanced automation in high-throughput screening platforms and affinity strategies, the paradigms for screening lead compounds have led to a shift toward more mechanism-based screening (6). For the first time, our group found that cardiac glycosides may be active compounds of *Adonis coerulea* in terms of its acaricidal activity using proteomics and surface plasmon resonance technology, which also is a sensitive and environmentally friendly analytical method (7). In addition, compared with the traditional empirical methods of screening lead compounds from the synthetic compound libraries (million compounds), traditional medicines with antiparasitic activity or natural products also have been attracting many people's interest as a way of finding new agents. More and more active compounds from plants or traditional medicines have been discovered, and the potential targets or mechanisms of action explored.

Our group established this Research Topic to present the development of antiparasitic activity and the modes of action of natural products and traditional medicines. Nine papers by 73 authors from nine different countries were published to perform the latest progress in their field, which will help us find more interesting topics. The anthelmintic activity against *Teladorsagia circumcincta* of Nordic bark extracts (Athanasiadou et al.) and *Diospyros anisandra* (Flota-Burgos et al.) are explained respectively, and the leishmanicidal activity of licochalcone A *in vitro* and in an experimental model of *Leishmania* (Leishmania) infantum was also proven (Souza et al.). S-Methylcysteine (SMC) presented the ameliorate the intestinal damage Induced by Eimeria tenella Infection (Elmahallawy et al.) and silver nanoparticles biosynthesized with *Salvia officinalis* leaf performed a protective effect on the hepatic tissue injury induced by *Plasmodium chabaudi* (Metwally et al.). Silva Bello et al. proved the prophylactic effects of ivermectin and closantel on *Oestrus ovis* infestation in sheep. Arfuso et al. and Li et al. also made contributions to this Research Topic. We are aware that the selection of papers is incomplete and does not do justice to the importance of the research field “*Antiparasitic Activity and the Modes of Action of Natural Products and Traditional Medicines*”. However, it does reflect to some extent where we currently are knowledge-wise, and the topic should receive more attention and be discussed by people.

Currently, the development of new veterinary drugs is slow worldwide. It is important to explore new ways of finding more rational and effective strategies to screen novel active compounds: a crucial but not easy task. The discovery of new and promising antiparasitic agents from natural products and traditional medicines will be important for controlling parasites in the future.

## Author Contributions

XS wrote the manuscript. AP and GI revised the manuscript. JZ supervised the manuscript. All authors contributed to the article and approved the submitted version.

## Funding

This work was financed by the National Natural Science Foundation of China (31772790) and the Innovation Project of the Chinese Academy of Agricultural Sciences (No. CAAS-ASTIP-2015-LIHPS).

## Conflict of Interest

The authors declare that the research was conducted in the absence of any commercial or financial relationships that could be construed as a potential conflict of interest.

## Publisher's Note

All claims expressed in this article are solely those of the authors and do not necessarily represent those of their affiliated organizations, or those of the publisher, the editors and the reviewers. Any product that may be evaluated in this article, or claim that may be made by its manufacturer, is not guaranteed or endorsed by the publisher.
